# Primary familial brain calcification presenting with parkinsonism and motor complications caused by a novel *SLC20A2* variant: a case report

**DOI:** 10.3389/fneur.2024.1382534

**Published:** 2024-07-05

**Authors:** Dandan Sun, Yu Wang, Jiawei Wang, Shijing Wang, Ling Zhu, Kun Xia, Yunyun Zhang, Xun Wang

**Affiliations:** ^1^Department of Graduate, Anhui University of Chinese Medicine, Hefei, China; ^2^Institute of Neurology, Anhui University of Chinese Medicine, Hefei, China; ^3^Department of Neurology, Yueyang Hospital of Integrated Traditional Chinese and Western Medicine, Shanghai University of Traditional Chinese Medicine, Shanghai, China

**Keywords:** primary familial brain calcification, Fahr’s disease, *SLC20A2* gene, parkinsonism, motor fluctuation, levodopa-induced dyskinesia

## Abstract

Primary familial brain calcification (PFBC), also known as Fahr’s disease, is a central nervous system calcium deposition disorder with symmetrical basal ganglia calcification. Most PFBC cases are caused by *SLC20A2* gene variant. We report a Chinese female patient with PFBC and dopamine-responsive parkinsonism who had motor fluctuations and dyskinesia and recovered effectively after symptomatic medication adjustment. A novel heterozygous missense variant was found by whole-exome sequencing and proven harmful by family validation and genetic analysis. This example expands the phenotype of *SLC20A2*-associated PFBC patients and shows the clinical efficacy of dopaminergic replacement treatment.

## Introduction

Primary familial brain calcification (PFBC), originally known as Fahr’s disease, is a hereditary neurodegenerative disorder characterized by multiple symmetrical intracranial calcifications. The main clinical features include a combination of various movement disorders and neuropsychiatric symptoms (1). Over the past 12 years, seven genes associated with PFBC pathogenesis have been identified (2), including four dominant (*SLC20A2*, *XPR1*, *PDGFB,* and *PDGFRB*) and three recessive (*MYORG*, *JAM2,* and *CMPK2*) genes. Variants in *SLC20A2* are the most common, with patients with PFBC with this allele having a higher probability of developing parkinsonism (1). However, unlike patients with Parkinson’s disease (PD), those with PFBC and a parkinsonism phenotype usually respond poorly to levodopa (3). Here, we report the case of a Chinese woman with PFBC due to a novel *SLC20A2* variant, who presented with parkinsonism while responding well to dopaminergic therapy, exhibiting symptom fluctuations and levodopa-induced dyskinesia during progression.

## Case presentation

**Figure 1:**
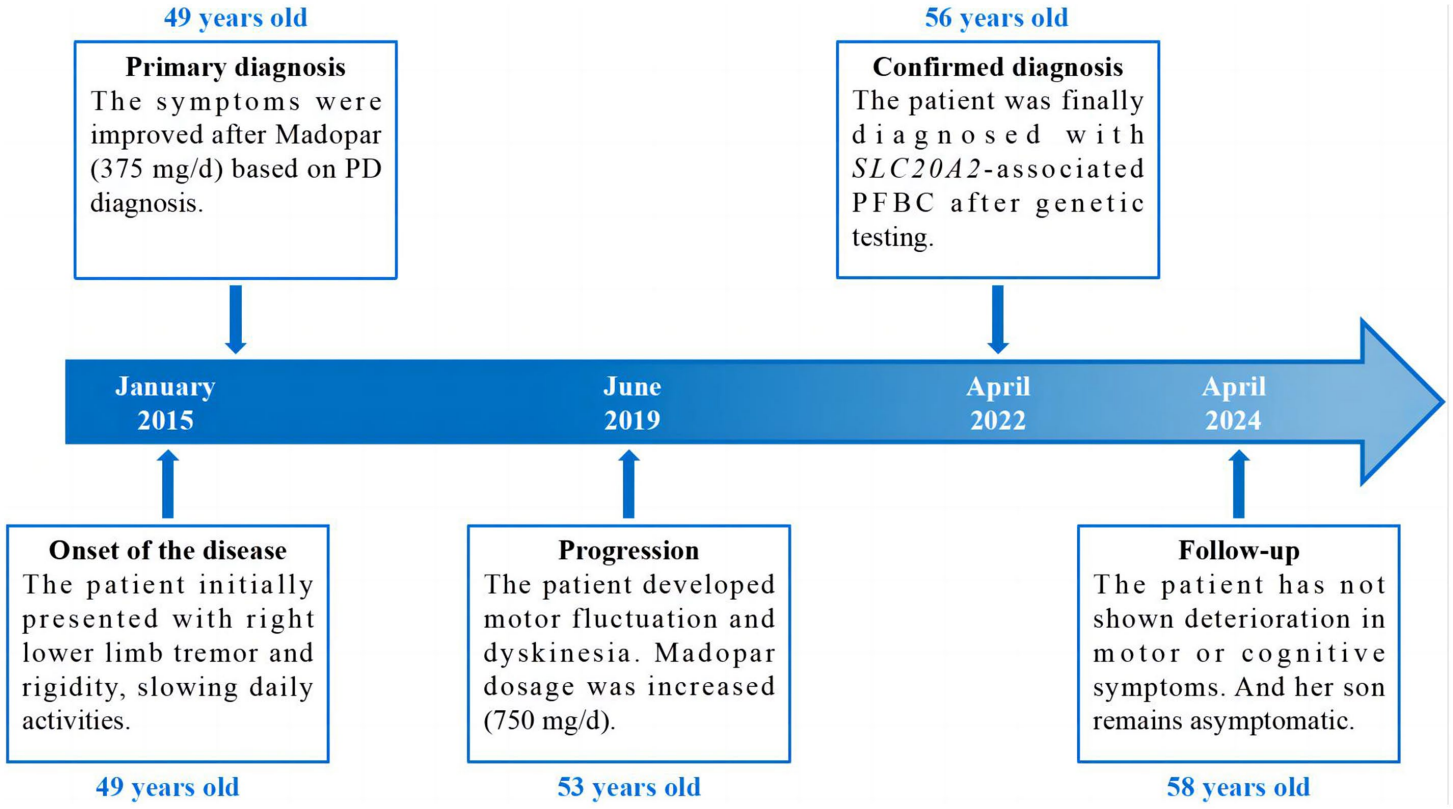
Timeline of the case report.

**Figure 2:**
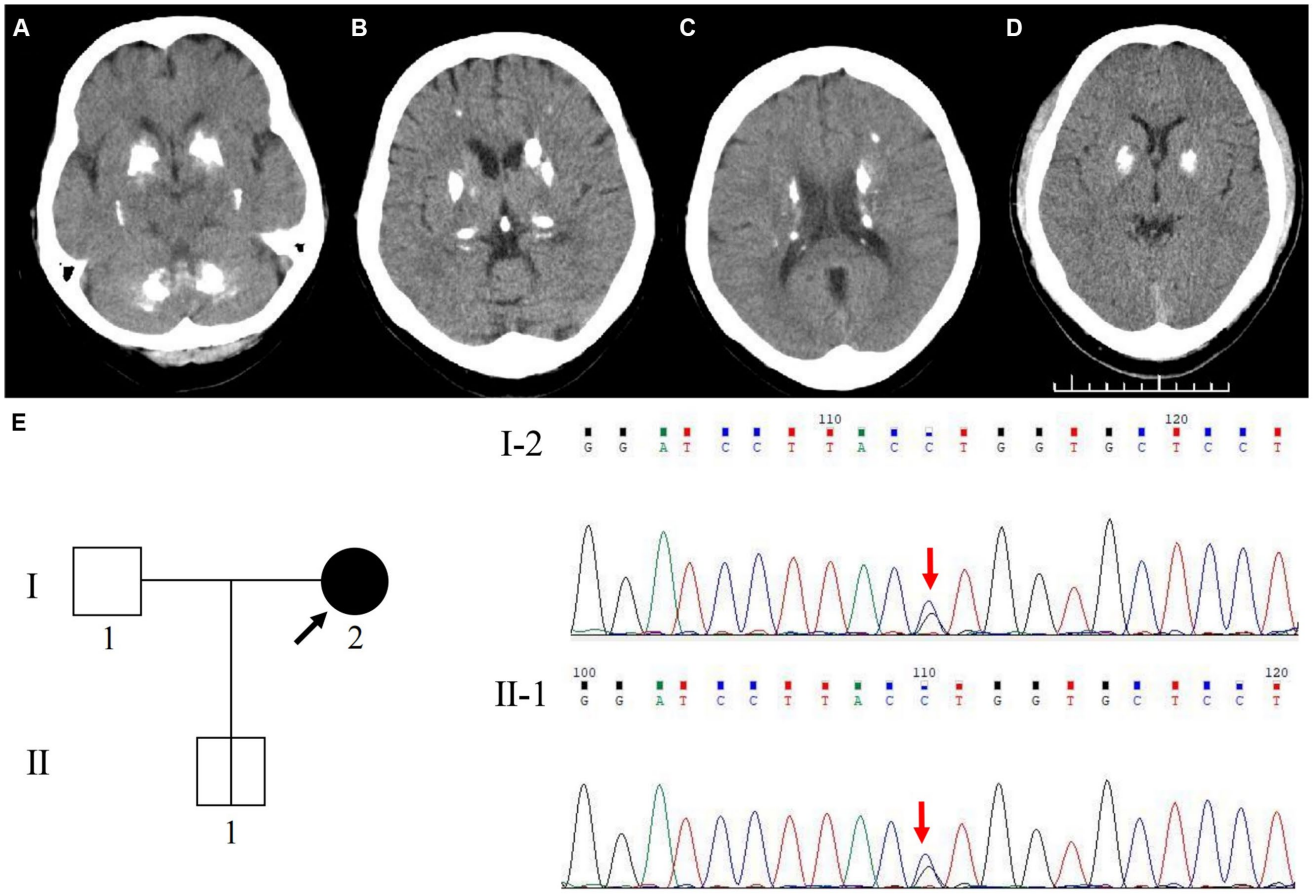
Cranial neuroimaging and Genetic analysis of *SLC20A2* in the family. Axial cranial CT scan shows symmetric hyperdensity lesions in the bilateral basal ganglia, cerebellar dentate nuclei, thalamus, periventricular region, and subcortical white matter of the parietal lobe, with widened sulci and mild brain atrophy of the proband **(A–C)**. Axial cranial CT scan shows symmetric hyperdensity lesions in the bilateral basal ganglia of the proband’s son **(D)**. WES identified a novel variant site in the *SLC20A2* gene, c.613G>C (p.Val205Leu). Genetic analysis revealed that the variant in the proband’s son (II-1) was inherited from his mother (I-2) **(E)**.

The proband (patient I-2) was a 56-year-old Chinese woman with slowly deteriorating extremity tremors, rigidity, and bradykinesia for 4 years, and involuntary head, neck, perioral, and trunk movements for 3 years (see the timeline in Figure 1). The patient had no history of stroke, brain injury, dopamine-blocking medication, toxic agent exposure, or familial inheritance. In 2015, the patient initially presented with a right lower limb static tremor and rigidity, slowing daily activities. The symptoms were improved after Madopar (375 mg/d) monotherapy based on PD diagnosis. In 2019, the patient experienced tremors in the right upper and left limbs, rigidity, bradykinesia, and gait freezing. Medication onset was delayed, and the ON phase was shortened; Madopar dosage was later increased (750 mg/d). The drug improved parkinsonism; however, the patient experienced involuntary head and trunk swinging, mouth licking, and shoulder shrugging. Levodopa-induced dyskinesia disappeared during the OFF phase. The patient was referred to our hospital in 2022. The patient had diminished facial expressions and no eye movement abnormalities, dysarthria, or dysphagia during the ON phase of the physical assessment. The patient trunk slanted forward, arm swing was decreased, while beginning and turning were delayed. No dysmetria or dizziness was observed upon standing up. With increasing muscular tension, the right side became heavier than the left. The left Babinski’s sign was positive. The score on the Mini-Mental State Examination (MMSE) was 22/30 (Corrected), while the score on the Montreal Cognitive Assessment (MoCA) was 20/30 (Corrected). The patient’s (Unified Parkinson’s Disease Rating Scale) UPDRS score during the “OFF” phase was 126. After adjusting the anti-parkinsonian medication regimen, the patient’s “ON” phase UPDRS score was 49, indicating a 61.1% improvement. Laboratory tests revealed normal liver function, electrolyte (including calcium and phosphorus metabolism), metal trace elements such as copper and iron, thyroid hormones and antibodies, parathyroid hormone, tumor indicators, and vitamin metabolism. Brain computed tomography (CT) revealed multiple symmetrical calcifications (Figures 2A–C). Whole-exome sequencing (WES) identified a c.613G>C (p. Val205Leu) variant in *SLC20A2*. Sanger sequencing confirmed that the proband’s son (patient II-1) had the same variant (Figure 2E), exhibiting symmetrical calcifications on brain CT (Figure 2D) despite lacking symptoms. The proband was diagnosed with *SLC20A2*-associated PFBC. We lowered the dose of madopar (375 mg/d) and added stalevo (325 mg/d) and pramipexole (0.75 mg/d). No significant disease progression was documented in outpatient follow-ups. The patient’s UPDRS score was 124 during the “OFF” phase (after a 12-h medication withdrawal) and 52 during the “ON” phase. Furthermore, the proband’s son continues to show no symptoms.

## Discussion

PFBC is a rare hereditary calcium deposition disorder of the central nervous system that often manifests in individuals aged 40–50, with an estimated prevalence of 0.1% (2). PFBC is clinically heterogeneous. A study of 516 patients identified a wide range of neurological and psychiatric symptoms, from common motor symptoms such as parkinsonism, dystonia, and tremors to rarer symptoms such as ataxia, seizures, headache, vertigo, and urinary tract symptoms (1). Our patient presented with parkinsonism and cognitive impairment. Based on neuroimaging evidence of calcification, PFBC was suggested. Finally, using genetic testing, a novel *SLC20A2* heterozygous missense variant was identified. This variant has not been reported in the gnomAD East Asian population database, ClinVar, or literature. The c.613G>C (p.Val205Leu) variant was predicted to be “Tolerated” by the SIFT software^1^ and “Benign” by the PolyPhen-2 software.^2^ Additionally, the proband’s son carried the same variant accompanied by brain calcification, suggesting a high concordance between the locus and clinical phenotype within the family, indicating it as a pathogenic variant. *SLC20A2* pathogenic variants were the first to be identified (4) and are the most common among Chinese patients (5). Up to 60.3% of symptomatic patients carry *SLC20A2* variants (1). *SLC20A2* encodes the type III sodium phosphate transporter 2 protein (PiT2), which is specifically expressed in the choroid plexus of the brain. *In vitro* experiments have demonstrated that variants can directly disrupt cerebral phosphate homeostasis, increasing the uptake of phosphate in the extracellular matrix and cerebrospinal fluid, which is currently believed to be the primary cause of PFBC (6). Moreover, based on pathological studies and analyses of calcification composition, calcifications are primarily deposited around the outer membrane cells of cerebral microvessels, blood vessel walls, adjacent neurons, and glial cells. Hence, elevated phosphate levels leading to disturbances in the neurovascular unit (NVU) and induction of cell ossification are considered significant aspects of the complex mechanisms underlying ectopic central calcification in PFBC (6).

Brain calcifications in patients with PFBC are entirely penetrant and display similar radiological features (7). Various degrees of other typical calcified brain regions, including the bilateral basal ganglia, particularly the globus pallidus, as well as other areas such as the subcortical white matter, cerebellar dentate nucleus, thalamus, and cortex, have also been reported. However, although our patient exhibited cerebellar lesions, no clinical cerebellar signs were observed, consistent with research findings that no definite correlation between the severity and location of calcifications and clinical phenotype exists (7). This phenomenon may be related to the lower rate of clinical manifestations observed with *SLC20A2* than with other PFBC causative genes (1). As the cooccurrence of *SLC20A2*-associated PFBC with dopamine-responsive parkinsonism is very unusual (3, 8–11), monitoring the dopamine responsiveness of the patient to assess the possible benefits of dopamine replacement therapy is essential. Notably, Yamada (10) and Manyam (11) discovered not only pathological calcification but also the presence of Lewy bodies in patients with dopamine-responsive PFBC. As PD is a prevalent neurodegenerative condition among older individuals, the possibility of comorbidity between PFBC and PD should not be overlooked. We were also intrigued by the unexpectedly favorable response to dopamine in this specific patient. Unfortunately, due to research facility limitations, we were unable to further explore the underlying neural mechanisms through measurements of striatal dopamine transporter uptake rate. This is a limitation of the paper.

PFBC is a slowly progressing neurodegenerative disease characterized by clinical heterogeneity and incomplete penetrance (6). Unexpected brain calcification seen on radiological imaging can be an initial diagnostic clue, even when clinical symptoms do not completely align (1). In the case report presented here, the proband’s son showed symmetrical basal ganglia calcification, but no clinical symptoms were observed during the two-year follow-up. However, it is speculated that as the proband’s son ages and intracranial calcification progresses, he may develop a phenotype similar to the proband in the future. Since there are currently no preventive or disease-modifying therapies for PFBC, it is important to enhance screening and follow-up for carriers within the family and provide genetic counseling.

In conclusion, this case confirms the clinical heterogeneity of PFBC associated with *SLC20A2* variants and provided new clinical evidence for PFBC diagnosis. In the absence of effective disease-modifying treatments and calcium chelation medications, we achieved desirable therapeutic results using a customized pharmacological strategy, indicating that symptomatic treatment has positive clinical implications for patients with PFBC presenting with the parkinsonism phenotype.

## Data availability statement

The datasets presented in this article are not readily available because of ethical and privacy restrictions. Requests to access the datasets should be directed to the corresponding authors.

## Ethics statement

The studies involving humans were approved by the Medical Research Ethics Committee of the Affiliated Hospital of the Neurology Institute of Anhui University of Chinese Medicine. The studies were conducted in accordance with the local legislation and institutional requirements. The participants provided their written informed consent to participate in this study. Written informed consent was obtained from the individual(s) for the publication of any potentially identifiable images or data included in this article.

## Author contributions

DS: Writing – original draft, Conceptualization, Data curation, Formal analysis. YW: Writing – original draft, Conceptualization, Data curation, Formal analysis. JW: Writing – original draft, Investigation, Methodology, Project administration. SW: Writing – original draft, Investigation, Methodology, Project administration. LZ: Writing – original draft, Investigation, Methodology, Project administration. KX: Writing – original draft, Investigation, Methodology, Project administration. YZ: Writing – review & editing, Supervision, Validation. XW: Writing – review & editing, Supervision, Validation.

## Footnotes

^1^ https://sift.bii.a-star.edu.sg/

^2^ http://genetics.bwh.harvard.edu/pph2/
